# The proposed EU Directives for AI liability leave worrying gaps likely to impact medical AI

**DOI:** 10.1038/s41746-023-00823-w

**Published:** 2023-04-26

**Authors:** Mindy Nunez Duffourc, Sara Gerke

**Affiliations:** 1 Penn State Dickinson Law, Carlisle, PA USA; 2 New York University Law School, New York, NY USA

**Keywords:** Health policy, Law

## Abstract

Two newly proposed Directives impact liability for artificial intelligence in the EU: a Product Liability Directive (PLD) and an AI Liability Directive (AILD). While these proposed Directives provide some uniform liability rules for AI-caused harm, they fail to fully accomplish the EU’s goal of providing clarity and uniformity for liability for injuries caused by AI-driven goods and services. Instead, the Directives leave potential liability gaps for injuries caused by some black-box medical AI systems, which use opaque and complex reasoning to provide medical decisions and/or recommendations. Patients may not be able to successfully sue manufacturers or healthcare providers for some injuries caused by these black-box medical AI systems under either EU Member States’ strict or fault-based liability laws. Since the proposed Directives fail to address these potential liability gaps, manufacturers and healthcare providers may have difficulty predicting liability risks associated with creating and/or using some potentially beneficial black-box medical AI systems.

## Introduction

On September 28, 2022, the European Commission (EC) published two proposed Directives that impact liability for artificial intelligence (AI): a proposed Product Liability Directive (PLD)^1^ and a proposed AI Liability Directive (AILD)^2^. In combination with the proposed Artificial Intelligence Act (AI Act)^3^, these newly proposed legal acts represent continued progress toward the European Union (EU)’s efforts to develop a unified approach to regulating the development and risks of AI technologies in the EU marketplace^4^. One difference to note, however, is that the AI Act is a proposed Regulation, which will be directly applicable in all EU Member States, while the PLD and AILD are proposed Directives, which would still need to be transposed into national law (TFEU, Art. 288)^5^. Once transposed into national law, the two proposed Directives will operate in conjunction with EU Member States’ existing national strict and fault-based liability laws to govern liability for AI-caused injuries. While the PLD generally forbids EU Member States from adopting national laws that are either more or less restrictive than those set forth in the Directive (Art. 3)^1^, the AILD generally allows them to adopt stricter national laws to govern non-contractual liability for AI-caused damages that fall outside of the PLD’s scope (Art. 1(4), Recital 11)^2^. As a result, the EU Member States still have considerable discretion in developing national rules to govern liability for injuries caused by AI systems.

According to the EC, however, the “[c]urrent national liability rules, in particular based on fault, are not suited to handling liability claims for damage caused by AI-enabled products and services” (AILD Explanatory Memorandum [providing reasons for the proposed Directive])^2^. Specifically, the EC recognized that some cases of AI-caused injury may fall into “compensation gaps” under national law, and thus may fail to provide victims with a level of liability protection comparable to what they would receive in similar cases not involving AI (AILD, Recital 4)^2,6^. As the EC felt that such compensation gaps would both decrease acceptance and trust in AI and introduce “legal uncertainty” surrounding liability for AI-caused injuries, it aimed to provide a uniform approach at the EU level to ensure liability protection for such injuries through the proposed Directives (AILD, Recitals 4, 6–8)^2^.

The proposed PLD’s strict liability rules and the proposed AILD’s fault-based liability rules make some progress toward the EC’s goal by (1) directly addressing unique risks posed by AI systems and (2) mitigating the information asymmetry by making it easier for claimants to prove their case. However, for some medical AI systems, the proposed Directives still likely do not ensure a path to recovery under national law, making it difficult for injured patients to successfully sue and also difficult for manufacturers and healthcare providers in the EU to predict liability risks associated with creating and/or using these systems in patient care (see AILD, Explanatory Memorandum)^2^. This is a significant concern considering that uncertainty surrounding liability “ranked amongst the top three barriers to the use of AI by European companies” (AILD, Explanatory Memorandum)^2^. Manufacturers and healthcare providers in the U.S. should also pay attention to the developing liability situation surrounding AI-caused injuries in the EU because the EU’s early approach may have implications for the similarly unclear liability situation for some medical AI systems in the U.S.

To give manufacturers and healthcare providers a sense of how EU Member States’ national laws might govern liability under the proposed PLD and AILD, we discuss two types of medical AI systems—i.e., autonomous and non-autonomous black-box medical AI. In particular, we reveal two main potential liability gaps that may occur when these systems cause a patient injury. We observe that unless these potential liability gaps are addressed at the EU level, Member States may, as predicted by the EC, seek to fill the gaps with non-harmonized national law, defeating the EC’s efforts to “reduce[] legal uncertainty of businesses developing or using AI [including healthcare providers] regarding their possible exposure to liability and prevent[] the emergence of fragmented AI-specific adaptations of national civil liability rules” (AILD, Explanatory Memorandum)^2^.

## Black-box medical AI

The EC recognizes that “[t]he specific characteristics of AI, including complexity, autonomy and opacity (the so-called “black box” effect), may make it difficult or prohibitively expensive for victims to identify the liable person and prove the requirements for a successful liability claim” (AILD, Explanatory Memorandum)^2^. Deep learning, a subset of AI that uses artificial neural networks to identify data patterns, is typically considered a black-box AI model because it is usually impossible for humans to understand its reasoning process^7,8^.

These days, black-box AI models are frequently “locked” when released on the market—meaning that they do not change and offer the same result every time the same input is applied to them^9^. However, their true potential is their capacity to be “adaptive” and continuously learn from new data^9^. Moreover, they can also be autonomous and make decisions without any human supervision. In medicine in particular, black-box AI models are used in most cases over interpretable models (“white boxes”) to strive for greater accuracy^10^. On the other hand, while black-box medical AI models such as deep learning models provide greater accuracy, they are considered noninterpretable, and thus their output can be unpredictable and not amenable to independent assessment by human healthcare providers^10,11^. The future liability situation for manufacturers and healthcare providers who create and/or use potentially beneficial black-box medical AI is unclear because injuries caused by a black-box AI’s output or failure to produce an output may still fall in a gap between liability for manufacturers and liability for healthcare providers, even with the newly proposed Directives.

## Potential liability gaps under national law with the proposed EU Directives

The newly proposed EU Directives—PLD and AILD—will likely fall short of providing a clear and uniform path to liability for some patients who are injured by some black-box medical AI systems. This is because patients may not be successful in lawsuits against either healthcare providers or manufacturers under national law, even with the newly proposed Directives. With regard to manufacturers, some black-box medical AI systems may (1) not be considered defective products subject to strict product liability under national law even with the proposed PLD’s rules *and* (2) cause an injury that cannot be connected to manufacturer fault subject to fault-based liability under national law even with the proposed AILD’s rules.

**Fig. 1:**
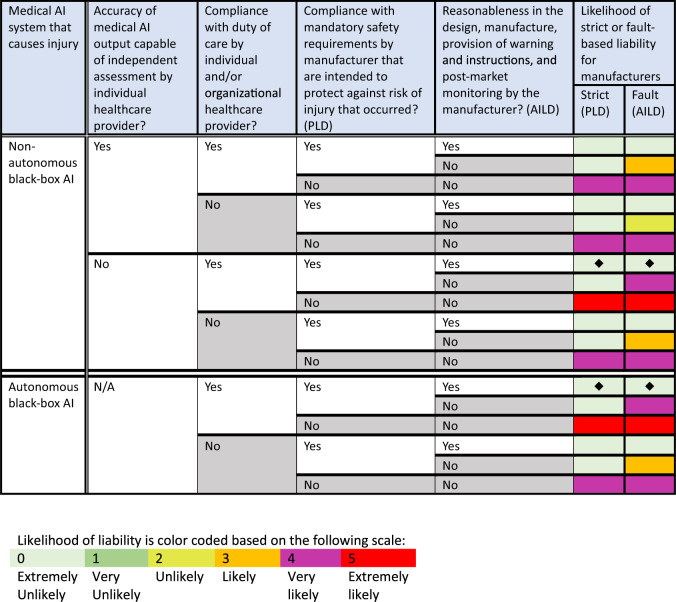
Likelihood of Manufacturers’ Future Liability under Member States’ National Law with Proposed PLD and AILD of 28 September 2022. Analysis of likelihood of potential liability for manufacturers of black-box medical AI systems that can cause injury (rows) based on variables related to the AI system’s features, use, and development (columns). Likelihood of liability is color coded based on the following scale: Light green box (0) = Extremely unlikely. Medium green box (1) = Very unlikely. Lime green box (2) = Unlikely. Orange box (3) = Likely. Purple box (4) = Very likely. Red box (5) = Extremely likely. Black diamonds represent gaps in liability when compared with likelihood of liability as shown in Fig. 2. *AI* artificial intelligence, PLD proposed Product Liability Directive, AILD proposed AI Liability Directive.

Figure 1 illustrates the likelihood of a successful lawsuit against manufacturers for patient injuries caused by some black-box medical AI systems under national law with the proposed PLD and AILD.

**Fig. 2:**
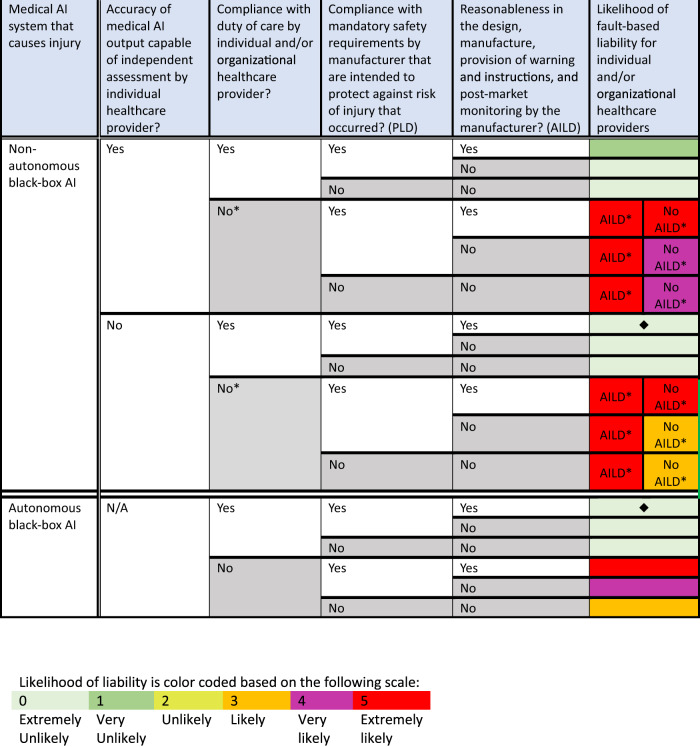
Likelihood of Healthcare Providers’ Future Liability under Member States’ National Law with Proposed PLD and AILD of 28 September 2022. Analysis of likelihood of potential liability for individual and organizational healthcare providers that use black-box medical AI systems that can cause injury (rows) based on variables related to the AI system’s features, use, and development (columns). Likelihood of liability is color coded based on the following scale: Light green box (0) = Extremely unlikely. Medium green box (1) = Very unlikely. Lime green box (2) = Unlikely. Orange box (3) = Likely. Purple box (4) = Very likely. Red box (5) = Extremely likely. Black diamonds represent gaps in liability when compared with likelihood of liability as shown in Fig. 1. The asterisk means that the AILD seems likely to aim to cover claims for damages if the non-compliance by the healthcare provider occurred prior to the AI output or failure to produce the AI output. However, the AILD does not appear to aim to cover claims for damages if the non-compliance by the healthcare provider occurred subsequent to the AI output (see AILD, Recital (15)). *AI* artificial intelligence, *PLD* proposed Product Liability Directive, AILD proposed AI Liability Directive.

With regard to healthcare providers, some black-box medical AI systems may cause an injury that also cannot be connected to the fault of either an individual or organizational healthcare provider under national law, even with the proposed AILD. Figure 2 illustrates the likelihood of a successful lawsuit against healthcare providers for patient injuries caused by some black-box medical AI systems under national law with the proposed PLD and AILD.

As shown in Figs. 1 and 2, there are two scenarios in which a patient’s lawsuit for an AI-caused injury against both manufacturers and healthcare providers is extremely unlikely to succeed. These scenarios, further explained in Supplementary Scenarios 1 and 2, possibly occur when (1) a non-autonomous black-box AI produces an output, which physicians review and rely upon but cannot independently assess because they cannot understand the AI’s algorithmic reasoning process (Supplementary Scenario 1), and (2) an autonomous black-box AI makes medical decisions not subject to independent physician review and assessment (Supplementary Scenario 2). As a result, these two scenarios demonstrate potential liability gaps for AI-caused patient injuries under national law, which are still not filled by the proposed PLD and AILD.

First, a patient in the EU who suffers an injury in either of the two supplementary scenarios is unlikely to succeed in a lawsuit against the manufacturer based on a defective product. The EC has already recognized that AI systems “with self-learning capabilities also raise the question of whether unpredictable deviations in the decision-making path can be treated as defects”^12^. Assume that in both scenarios, the hypothetical black-box AI systems have obtained an EU CE mark to certify that they have “been assessed by the manufacturer and deemed to meet EU safety, health and environmental protection requirements”^13^. If we further assume that the manufacturer complied with mandatory safety requirements (PLD, Art. 6(1)(f)) and that patients were properly informed that the AI was being used in their care and the conditions and risks associated with its use (see PLD, Art. 6(1)(h)), these black-box medical AI systems may not be considered defective products subject to strict liability for manufacturers under national law because the AI’s noninterpretable reasoning process may be deemed outside of the manufacturer’s control (PLD, Art. 6(1)(e))^1^.

We note that although some EU Member States may impose strict liability for “dangerous activity” outside of national product liability law, according to the EC, such liability “for the operation of computers, software or the like is so far widely unknown in Europe” and thus does not provide a clear path for recovery by patients who are injured by the black-box medical AI systems discussed here^12^.

Second, a patient in the EU who suffers an injury in either of the two scenarios is also unlikely to succeed in a lawsuit based on the fault of the manufacturer or the individual/organizational healthcare provider because the patient may not be able to identify a breach of a duty of care by any party. Notably, the AILD does not create any new substantive duties of care for manufacturers or individual/organizational healthcare providers, but rather relies on existing “dut[ies] of care under Union or national law” to govern “fault” (see AILD, Explanatory Memorandum and Recital (23))^2^. Generally, a party breaches their duty of care under national law if they fail to act reasonably under the circumstances (or fail to comply with the standard of care)^12^. The AILD’s reliance on existing duties of care to assess fault is problematic because, as the EC previously observed, “[t]he processes running in AI systems cannot all be measured according to duties of care designed for human conduct” making it difficult to determine fault^12^.

Patients injured by black-box AI systems may have trouble succeeding in lawsuits based on the fault of manufacturers or healthcare providers because, according to the EC, “[e]merging digital technologies make it difficult to apply fault-based liability rules, due to the lack of well established models of proper functioning of these technologies and the possibility of their developing as a result of learning without direct human control”^12^. For example, a manufacturer may not be at fault if the patient’s injury is not caused by the manufacturer’s design of a black-box AI system, but rather by “subsequent choices made by the AI technology independently”^12^. Similarly, assuming that the mere use of an EU CE-marked black-box AI system does not breach a duty of care, individual/organizational healthcare providers may not be at fault for AI-caused damage if the providers lack both control over the AI’s learning and reasoning processes and the ability to independently assess the accuracy of the AI’s output. As a result, if neither the manufacturer nor the healthcare provider is likely to face liability for AI-caused damage, as demonstrated in Supplementary Scenarios 1 and 2, under national law, even with the newly proposed Directives, two potential liability gaps emerge.

To further illustrate how these potential gaps might manifest in practice, we present a hypothetical example of each type of system. The first type is a non-autonomous black-box AI that predicts the origin of metastatic cancer of unknown primary (CUP) by “utilis[ing] [a] large number of genomic and transcriptomic features”^14^. As cancer origin “can be a significant indicator of how the cancer will behave clinically,” a human physician might rely on the AI’s prediction to develop a treatment plan^14^. The second type is an autonomous black-box AI that uses a deep learning algorithm to evaluate X-rays without the input of a radiologist^15^. This AI can generate final radiology reports for X-rays that reveal no abnormalities, while those with suspected abnormalities will be referred to a human radiologist for final evaluation and reporting^15^.

Assume that in both hypotheticals, in addition to complying with all safety requirements and obtaining an EU CE marking, the black-box AI systems are functioning as designed by the manufacturer (i.e., to make decisions and recommendations using noninterpretable complex algorithmic reasoning), their design was reasonable under the circumstances, and the manufacturer was reasonable in providing warnings, instructions, and post-market monitoring. In this case, the injured patient is not likely to be successful in a lawsuit against the manufacturer based on either a product defect or manufacturer fault. Additionally, if the healthcare organizations and individual providers involved also complied with their duties of care associated with using the hypothetical black-box AI systems, for example through the reasonable selection and implementation of the AI system in clinical practice, the patient is not likely to be successful in a lawsuit against the healthcare providers based on provider fault.

As shown in Supplementary Scenario 1, Potential Liability Gap 1 will manifest for the first hypothetical black-box system when the non-autonomous AI provides an incorrect CUP prediction that causes a patient injury. This liability gap occurs because while a human physician oversaw and relied upon the AI’s prediction to maximize the patient’s chance for successful treatment, they could not independently assess the accuracy of the AI’s output. As a result, the physician’s reliance on the output of a system used in accordance with its EU CE marking will likely not violate an applicable duty of care for the physician who could not have known that the AI’s prediction was incorrect. This gap in liability is remarkable because the AI is making a medical recommendation that would have otherwise been made by a human physician, who the patient could have sued for fault (or medical malpractice). For example, had a human pathologist misclassified the CUP, the patient would likely succeed in a lawsuit against the human pathologist if their misclassification violated the standard of care or *medical lege artis*. However, this path to liability becomes unavailable under national law even with the proposed AILD when a non-autonomous black-box AI, rather than a human pathologist, predicts the CUP origin, undermining the EC’s goal of providing the same level of protection for victims of AI-caused damage.

As shown in Supplementary Scenario 2, Potential Liability Gap 2 will manifest for the second hypothetical black-box AI system when the autonomous AI reports no abnormality for an X-ray film with remarkable findings, which leads to a missed diagnosis and patient injury. This gap in liability is remarkable because the AI is making a medical decision that would have otherwise been made by a human physician, who the patient could have sued for fault (or medical malpractice). For example, if a human radiologist missed an abnormality in the X-ray film, the patient would likely succeed in a lawsuit against the radiologist if the missed abnormality violated the standard of care or *medical lege artis*. However, this path to liability becomes unavailable under national law even with the proposed AILD when an autonomous black-box AI, rather than a human radiologist, evaluates the X-ray, thus again undermining the EC’s goal of providing the same level of protection for victims of AI-caused damage.

## Implications of the potential liability gaps

The proposed PLD and AILD fail to meet the EC’s goal of filling “compensation gaps” to provide a clear approach to liability in cases when black-box medical AI causes damage solely as a result of its complex, opaque, and/or autonomous reasoning process. This is because absent a product defect covered by the proposed PLD or manufacturer fault upon which to base manufacturer liability (see Fig. 1) or fault by an individual or organizational healthcare provider upon which to base healthcare provider liability (see Fig. 2), there may be no national liability law to compensate for damage caused by black-box medical AI (see Supplementary Scenarios 1 and 2). Crucially, the potential liability gaps that we have identified for some black-box medical AI systems represent scenarios in which victims would have had more legal protection without AI involvement in their medical care.

These potential gaps in liability for AI-caused damage thwart the EU’s goals of enhancing public trust in AI and “increasing legal certainty through harmonized measures at EU level, compared to possible adaptations of liability rules at national level” (AILD, Explanatory Memorandum)^2^. Instead, as the EC suspected, without a clear and uniform path to liability, EU Member States may seek to fill these gaps through national liability laws, “leading to different levels of protection and distorted competition among businesses from different Member States” (AILD, Explanatory Memorandum)^2^. As a result, without the ability to reasonably predict liability risks, manufacturers may opt out of creating and healthcare providers may opt out of using potentially beneficial black-box medical AI systems. For now, manufacturers who create black-box medical AI systems should comply with all mandatory safety requirements and follow industry best practices. Healthcare providers who use AI systems to treat patients should document compliance with all recommended safety requirements related to the system’s implementation and use in patient care. Further, individual providers who rely on recommendations from black-box AI systems should strive to independently assess the AI’s output when possible and carefully document their reasons for relying on or departing from the AI’s recommendation when independent assessment is not possible.

Finally, we note a comparable situation in the U.S., where liability for injuries caused by some black-box medical AI systems will fall in a gap between product liability law for manufacturers and fault-based medical liability law for healthcare providers because there is no natural or legal person that will be responsible under existing U.S. tort law for injuries caused solely by the AI system’s noninterpretable reasoning process [16]. As a result, both AI manufacturers and healthcare providers in the U.S. can learn valuable lessons about potential liability for black-box medical AI systems by closely monitoring the new developments in the EU.

## Conclusion

The EC is certainly making commendable progress in recognizing, evaluating, and addressing liability problems caused by AI systems. However, its goal of reducing fragmentation of national laws governing AI liability to provide stakeholders with certainty about the legal situation is not fully accomplished by the proposed PLD and AILD if injuries caused by black-box medical AI are not covered under either EU Member States’ strict or fault-based product liability laws for manufacturers or fault-based medical liability laws for healthcare providers. Only when the EC identifies and implements additional measures to fill the remaining potential liability gaps for black-box medical AI will the EU enjoy “[h]armonised measures at EU level [that]… significantly improve conditions for the rollout and development” of black-box medical AI systems (AILD, Explanatory Memorandum)^2^.

## Supplementary information

Supplementary Information

## Data Availability

This article does not make direct use of any data.
