# Supplementary material for: The proposed EU Directives for AI liability leave worrying gaps likely to impact medical AI
Source: NPJ Digit Med. 2023 Apr 26;6:77. doi: 10.1038/s41746-023-00823-w (PMC10133336; doi:10.1038/s41746-023-00823-w)
Supplement: Supplementary file 1 — Supplementary Information [file 41746_2023_823_MOESM1_ESM.pdf]

Supplementary Scenarios

Supplementary Scenario 1: In this scenario, an EU CE-marked non-autonomous black-box AI system's recommendation (output) caused a patient injury [Column 1]. Because the system is a black box, the basis for its recommendation is not knowable (noninterpretable). Because the system is non-autonomous, its recommendation is reviewed by an individual healthcare provider, who then relies on the recommendation to make a subsequent medical decision affecting the patient's care. However, the accuracy of the AI's recommendation cannot be independently assessed by the individual healthcare provider because they cannot understand the AI's algorithmic reasoning process [Column 2]. As a result, the individual provider's reliance on the AI's recommendation will likely not breach a duty of care because the provider could not have known that the AI's recommendation was wrong. The healthcare organization and/or individual provider that chose to use the AI in the patient's care was not negligent in its implementation, use, and/or oversight of the AI and thus complied with all duties of care under national fault-based medical liability law [Column 3]. Because there is no failure to comply with a duty of care (no fault), the proposed AILD will *not* apply in the national fault-based medical liability law applicable to healthcare providers. Additionally, the manufacturer of the AI system complied with all mandatory safety requirements intended to protect against the patient injury that occurred in this case, so the AI system's wrong recommendation may not be deemed a defect under the proposed PLD [Column 4]. As a result, the proposed PLD will likely not apply in the national strict product liability law applicable to manufacturers. Finally, because the manufacturer was reasonable in the design and manufacture and the provision of warnings, instructions, and after-market monitoring, there is likely no manufacturer fault and the proposed AILD will not apply in the national fault-based product liability law applicable to manufacturers [Column 5]. Any claim for injury caused by the AI recommendation in this case will be judged under national liability law without the proposed AILD or PLD, and liability against the healthcare organization, individual provider, and/or manufacturer is *extremely unlikely* because there is neither a product defect nor a legally responsible party at fault [Column 6]. This scenario represents **Liability Gap 1**.

[1] Medical AI system that causes injury	[2] Accuracy of medical AI output capable of independent assessment by individual healthcare provider?	[3] Compliance with duty of care by individual and/or organizational healthcare provider?	[4] Compliance with mandatory safety requirements by manufacturer that are intended to protect against risk of injury that occurred? (PLD)	[5] Reasonableness in the design, manufacture, provision of warning and instructions, and post-market monitoring by the manufacturer? (AILD)	[6] Likelihood of strict or fault-based liability for manufacturers and likelihood of fault-based liability for individual/ organizational healthcare providers under Member States' national law with proposed PLD and AILD of 28 September 2022
Non-Autonomous black-box AI	No	Yes	Yes	Yes	Extremely Unlikely<br>**Liability Gap 1**

AI = artificial intelligence
PLD = proposed Product Liability Directive
AILD = proposed AI Liability Directive

Supplementary Scenario 2: In this scenario, an EU CE-marked autonomous black-box AI system's decision (output or failure to produce an output) caused a patient injury [Column 1]. Because the system is a black box, the basis for its decision is not knowable (noninterpretable). Because the system is autonomous, its decision is not reviewed by an individual healthcare provider, and there is also no opportunity for an individual healthcare provider to independently assess the accuracy of the AI's decision [Column 2]. The healthcare organization and/or individual provider that chose to use the AI in the patient's care was not negligent in its implementation, use, and/or oversight of the AI and thus complied with all duties of care under national fault-based medical liability law [Column 3]. Because there is no failure to comply with a duty of care (no fault), the proposed AILD will *not* apply in the national fault-based medical liability law applicable to healthcare providers. Additionally, the manufacturer of the AI system complied with all mandatory safety requirements intended to protect against the patient injury that occurred in this case, so the AI system's wrong decision may not be deemed a defect under the proposed PLD [Column 4]. As a result, the proposed PLD will likely not apply in the national strict product liability law applicable to manufacturers. Finally, because the manufacturer was reasonable in the design and manufacture and the provision of warnings, instructions, and after-market monitoring, there is likely no manufacturer fault and the proposed AILD will not apply in the national fault-based product liability law applicable to manufacturers [Column 5]. In this scenario, any claim for injury caused by the AI decision will be judged under national liability law without the proposed AILD or PLD, and liability of the healthcare organization, individual provider, and/or manufacturer is *extremely unlikely* because there is neither a product defect nor a legally responsible party at fault [Column 6]. This scenario represents **Liability Gap 2**.

[1] Medical AI system that causes injury	[2] Accuracy of medical AI output capable of independent assessment by individual healthcare provider?	[3] Compliance with duty of care by individual and/or organizational healthcare provider?	[4] Compliance with mandatory safety requirements by manufacturer that are intended to protect against risk of injury that occurred? (PLD)	[5] Reasonableness in the design, manufacture, provision of warning and instructions, and post-market monitoring by the manufacturer? (AILD)	[6] Likelihood of strict or fault-based liability for manufacturers and likelihood of fault-based liability for individual/ organizational healthcare providers under Member States' national law with proposed PLD and AILD of 28 September 2022
Autonomous black-box AI	N/A	Yes	Yes	Yes	Extremely Unlikely<br>**Liability Gap 2**

AI = artificial intelligence
PLD = proposed Product Liability Directive
AILD = proposed AI Liability Directive
